# *Chinese Wheat Mosaic Virus*-Induced Gene Silencing in Monocots and Dicots at Low Temperature

**DOI:** 10.3389/fpls.2018.01627

**Published:** 2018-11-14

**Authors:** Jian Yang, Tian-Ye Zhang, Qian-Sheng Liao, Long He, Juang Li, Heng-Mu Zhang, Xuan Chen, Jing Li, Jin Yang, Jin-Bang Li, Jian-Ping Chen

**Affiliations:** ^1^Institute of Plant Virology, Ningbo University, Ningbo, China; ^2^College of Life Science, Zhejiang SCI-Tech University, Hangzhou, China; ^3^State Key Laboratory Breeding Base for Zhejiang Sustainable Pest and Disease Control, Key Laboratory of Plant Protection and Biotechnology, Ministry of Agriculture, Beijing, China; ^4^Zhejiang Provincial Key Laboratory of Plant Virology, Institute of Virology and Biotechnology, Zhejiang Academy of Agricultural Sciences, Hangzhou, China; ^5^Nanyang Academy of Agricultural Sciences, Nanyang, China

**Keywords:** RNA virus, gene silecning, *Chinese wheat mosaic virus*, vector, low temperature

## Abstract

Virus-induced gene silencing (VIGS) is an important tool for functional genomics studies in plants. With this method, it is possible to target most endogenous genes and downregulate the messenger RNA (mRNA) in a sequence-specific manner. *Chinese wheat mosaic virus* (CWMV) has a bipartite, single-strand positive RNA genome, and can infect both wheat and *Nicotiana benthamiana*, and the optimal temperature for systemic infection in plants is 17°C. To assess the potential of the virus as a vector for gene silencing at low temperature, a fragment of the *N. benthamiana* or wheat phytoene desaturase (PDS) gene was expressed from a modified CWMV RNA2 clone and the resulting photo bleaching in infected plants was used as a reporter for silencing. Downregulation of PDS mRNA was also measured by quantitative reverse-transcriptase polymerase chain reaction (RT-qPCR). In experiments using fragments of PDS ranging from 500 to 1500 nucleotides, insert length influenced the stability and the efficiency of VIGS. The CWMV induced silencing system was also used to suppress miR165/166 and miR3134a through expression of miRNA target mimics. The relative expression levels of mature miR165/166 and miR3134a decreased whereas the transcript levels of their target genes increased. Interestingly, we also found the CWMV-induced silencing system was more efficient compare with the vector based on *Barley stripe mosaic virus* (BSMV) or *Foxtail mosaic virus* (FoMV) in wheat or the vector based on TRV in *N. benthamiana* at 17°C. In summary, the CWMV vector is effective in silencing endogenous genes and miRNAs at 17°C, thereby providing a powerful tool for gene function analysis in both *N. benthamiana* and wheat at low temperature.

## Introduction

Virus-induced gene silencing (VIGS) is a particularly powerful tool for studying plant functional genomics, especially for those organisms where genetic transformation is difficult (Burch-Smith et al., 2004; Robertson, 2004; Bernacki et al., 2010; Barciszewska-Pacak et al., 2016). VIGS is based on the plant’s endogenous RNA defense response that recognizes the accumulation of foreign double-stranded RNAs (dsRNA) and targets these sequences for degradation (Ding, 2000). In brief, the viral genome is used as a vector to carry an inserted sequence fragment of the plant target gene for silencing. Many plant viruses multiply in their hosts using a dsRNA intermediate, which is recognized as foreign dsRNA by the host endogenous RNA defense systems. This dsRNA, including the inserted target gene in VIGS studies, is then processed into the RNA interference pathway to degrade the complementary sequence (Denli and Hannon, 2003).

Many plant virus vectors have been developed for transient gene expression or silencing in plants (Pignatta et al., 2007; Igarashi et al., 2009; Jupin, 2013; Li and Yoshikawa, 2015). So far, more than 30 viruses have been reported to have been applied as VIGS vectors. These include *Tobacco mosaic virus* (TMV) (Velasquez et al., 2009), *Tobacco rattle virus* (TRV) (Ratcliff et al., 2001), and *Potato virus X* (PVX) (Himber et al., 2003) used for gene silencing in *Nicotiana benthamiana* and tomato. Most of VIGS vectors are applied in dicots, very little in monocots, especially rarely in wheat plant. To date, only two RNA viruses have been modified as vectors for VIGS in wheat plants, of which *Barley stripe mosaic virus* (BSMV)-based VIGS has been used as a vector in wheat, barley, oats, *N. benthamiana*, and *Brachypodium* (Pacak et al., 2010; Yuan et al., 2011; Scofield and Brandt, 2012) and *Foxtail mosaic virus* (FoMV)-based VIGS has been applied for functional genomics in barley, wheat, and foxtail millet (*Setaria italic*) (Liu et al., 2016). BSMV-based VIGS or TRV-based VIGS was widely applied in study gene function of wheat as well in many dicots, respectively. However, there have been no reports demonstrated effective VIGS vectors used for gene silencing at low temperature. Low temperature is an important environmental factor that affects the growth and development of plant. About 15–20°C is the optimal temperature for wheat growing. Therefore, it is important to develop a convenient and efficient VIGS vector suitable for gene silencing in both monocots and dicots, especially important for wheat.

Virus-induced gene silencing approaches have also been exploited to effectively block selected plant microRNA (miRNA) activity *in vivo*. Plant miRNAs are important in controlling plant development and responses to abiotic or biotic stress (Phillips et al., 2007; Sunkar et al., 2007; Simon and Meyers, 2011). Increasing numbers of miRNAs are being identified or predicted from large data sets generated by high-throughput sequencing but have unknown biological functions (Nobuta et al., 2010). Their functions are now being explored using approaches based on VIGS, including miRNA mimicry (MIM) (Franco-Zorrilla et al., 2007), artificial miRNA directed silencing of miRNA precursors (Eamens et al., 2011), transcriptional gene silencing of miRNA gene promoters (Vaistij et al., 2010), and short tandem target mimic (STTM) (Yan et al., 2012). STTM was first reported in *Arabidopsis* and is based on the expression of two short mimicking small RNA target sites separated by a size-optimized linker that is then degraded by small RNA degrading nucleases (Yan et al., 2012).

*Chinese wheat mosaic virus* is a member of the genus *Furovirus*, family *Virgaviridae* (Adams et al., 2009). Furoviruses naturally infect cereal plants and are transmitted by an obligate root infecting fungus-like organism, *Polymyxa graminis*. Like all known furoviruses, CWMV has a bipartite single-strand positive RNA genome (Diao et al., 1999; Ye et al., 1999; Yang et al., 2001). RNA1 (7147 nt) encodes three proteins required for viral replication and movement while RNA2 (3564 nt) is predicted to encode four proteins: the major capsid protein (CP, 19 kDa), two minor CP related proteins (N-CP, 23 kDa; CP-RT, 84 kDa) produced by translation initiation at a non-canonical CUG start codon or occasional read-through of the UGA termination codon, respectively, and a cysteine-rich RNA silencing suppressor (CRP, 19 kDa) (Diao et al., 1999; Yang et al., 2001; Andika et al., 2013; Sun et al., 2013). Recently, we constructed full-length cDNA clones of CWMV which were infectious in both wheat and *N. benthamiana* and determined that the optimal temperature for systemic infection of CWMV was 17°C (Yang et al., 2016).

In this study, we developed a CWMV-based VIGS vector that silenced the phytoene desaturase (PDS) gene at 17°C in both monocot (wheat) and dicot (*N. benthamiana*) hosts more effectively than a vector based on BSMV, FoMV, or TRV. We also demonstrate that the CWMV vector is effective in silencing endogenous miRNAs using STTM. Thus, the new vector is an economical and convenient tool that can be used to knockdown miRNAs and verify the function of “genes-of-interest” in plants in a low temperature environment.

## Results

### Infectivity Assay of Binary Constructs

**FIGURE 1:**
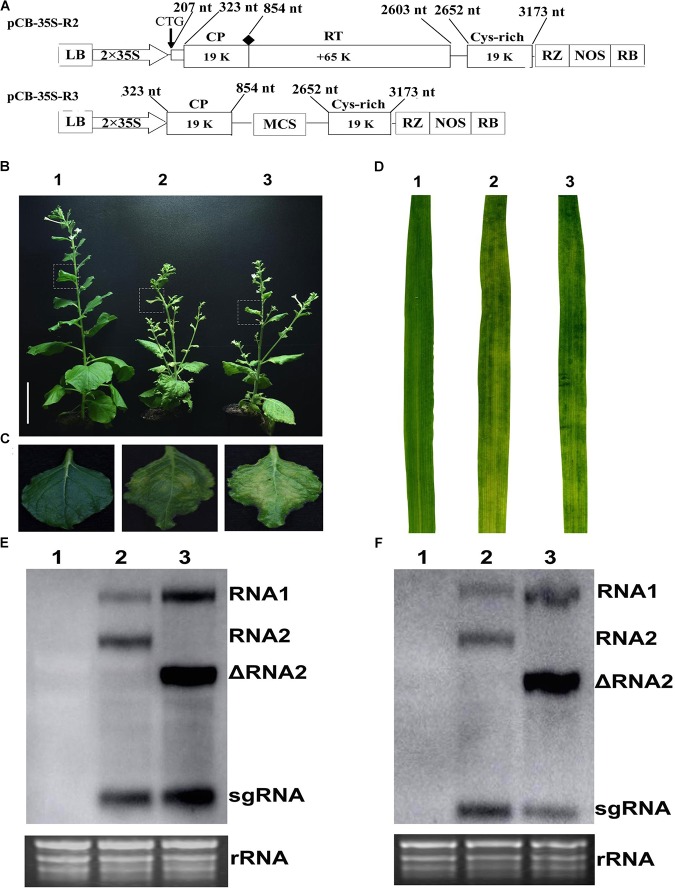
Infiltration assay using a mixture of the recombinant binary constructs pCB-35S-R1 and pCB-35S-R3. **(A)** Schematic diagram of the pCB-35S-R3 derived from binary plasmid pCB-35S-R2. pCB-35S-R3, the CTG initiation codon for N-CP was replaced with CAG, the wild-type codon CGG was replaced with TAG termination codon for CP and the region of nt 1098–2540 was replaced with MCS. **(B)** Mild green mosaic lesions formed on *N. benthamiana* leaves 54 days after inoculation. Lane 1, Mock-inoculated *N. benthamiana*; Lane 2, plant inoculated with wild-type CWMV; Lane 3, plant inoculated with ᐃCWMV. **(C)** Enlargement of the boxed part of **(B)**. **(D)** Symptoms on *T. aestivum* leaves 6 weeks after inoculation. Leave 1, mock-inoculated; Leave 2, inoculated with the sap from the leaves of *N. benthamiana* plant infected by wild-type CWMV; Leave 3, inoculated with the sap from the leaves of *N. benthamiana* plant infected by ᐃCWMV. **(E)** Northern blotting analysis. Lane 1, the total RNA extracted from the mock inoculated *N. benthamiana*; Lane 2, the total RNA extracted from the CWMV inoculated *N. benthamiana*; Lane 3, the total RNA extracted from the ᐃCWMV inoculated *N. benthamiana*. **(F)** Northern blotting analysis. Lane 1, the total RNA extracted from the mock inoculated *T. aestivum*; Lane 2, the total RNA extracted from the CWMV-inoculated *T. aestivum*; Lane 3, the total RNA extracted from the ᐃCWMV-inoculated *T. aestivum*. Ethidium bromide (EtBr)-stained rRNAs are shown as loading controls. sgRNA, subgenomic RNA; ᐃRNA2, the shorter form of RNA2.

This study used the full-length cDNA construct of CWMV RNA1 (pCB-35S-R1) described previously (Yang et al., 2016) together with modified constructs of RNA2 (ᐃRNA2). The earlier RNA2 construct (pCB-35S-R2) was modified to abolish the translation of the N-terminal extension of CP, to delete the entire RT domain of the CP and to insert a multiple cloning site (MCS) by PCR-based site-directed mutagenesis. This modified construct was designated it as pCB-35S-R3 (Figure 1A). The two constructs pCB-35S-R1 and pCB-35S-R3 were individually transformed into *Agrobacterium tumefaciens* strain GV3101 by electroporation and used to infiltrate leaves of *N. benthamiana*. By 54 days, the upper leaves of plants inoculated with ᐃCWMV including pCB-35S-R1 and pCB-35S-R3 developed numerous chlorotic local lesions similar to the plants inoculated with wild-type CWMV (Figures 1B,C). Following the method described using BSMV (Yuan et al., 2011), the infiltrated *N. benthamiana* leaf sap was then used to inoculate wheat. At 40–42 dpi, the newly-emerged leaves of inoculated plants consistently developed typical mosaic symptoms similar to those inoculated with sap containing wild-type virus (Figure 1D). Northern blot analysis confirmed that a shorter form of RNA2 could be detected in the upper leaves of both wheat and *N. benthamiana* when the modified construct had been used (Figures 1E,F). The results demonstrate successful and typical infection of both *N. benthamiana* and wheat using the modified RNA construct.

### CWMV-Induced Gene Silencing in *N. benthamiana* and Wheat

**FIGURE 2:**
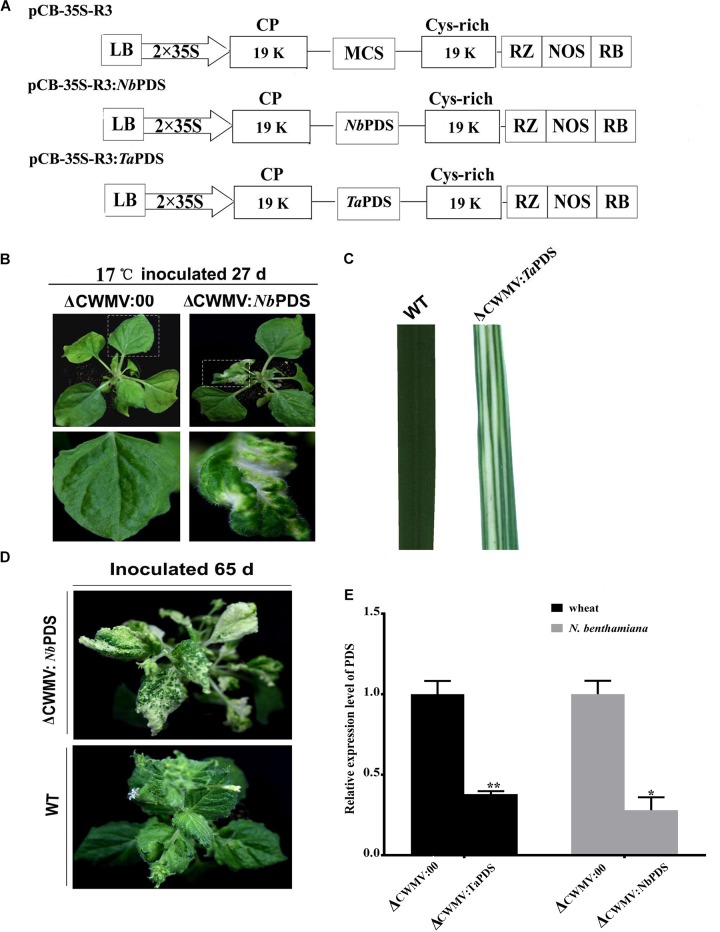
Evaluation of CWMV VIGS in *N. benthamiana* and *T. aestivum*. **(A)** Schematic diagram of the pCB-35S-R3:*Nb*PDS or pCB-35S-R3:*Ta*PDS derived from binary plasmid pCB-35S-R3. pCB-35S-R3:*Nb*PDS contains partial fragment of the PDS gene of *N. benthamiana* in the MCS site of pCB-35S-R3. pCB-35S-R3:*Ta*PDS contains partial fragment of the PDS gene of *T. aestivum* in the MCS site of pCB-35S-R3. **(B)** Photobleaching on *N. benthamiana* leaves 27 days after inoculation. **(C)** Photobleaching on *T. aestivum* leaves 30 days after rub-inoculation. **(D)** Photobleaching on *N. benthamiana* leaves 65 days after inoculation. **(E)** Expression levels of PDS transcripts in *T. aestivum* (*Ta*PDS) and *N. benthamiana* (*Nb*PDS). Bars represent the standard errors of the means. Three sample unequal variance directional *t*-test (^∗^*p* ≤ 0.05) was used to test the significance of the difference. The BSMV induced PDS silencing in *N. benthamiana* and *T. aestivum* served as controls. WT indicates wild-type plant. ^∗∗^Indicates significant difference at *P* ≤ 0.01.

*Nicotiana benthamiana* is the most widely used experimental host plant for VIGS because of its susceptibility to many plant viruses and the rapidity with which visible phenotypes appear (Goodin et al., 2008). To assess silencing by *Agrobacterium* mediated CWMV VIGS, partial PDS gene sequences derived from *N. benthamiana* (300 bp; nt 200–500 of EU165355) and *T. aestivum* (300 bp; nt 1025–1325 of FJ517553) were inserted into pCB-35S-R3 in the sense orientation to generate pCB-35S-R3:NbPDS and pCB-35S-R3:TaPDS, respectively (Figure 2A). These were co-infiltrated with the RNA1 construct to four-leaf stage *N. benthamiana* plants which were then maintained at 17°C. The ᐃCWMV:NbPDS (contains pCB-35S-R1 and pCB-35S-R3:NbPDS) caused systemic infection and induced visible photobleaching in the non-inoculated leaves of *N. benthamiana* at 27 dpi (Figure 2B). Photobleaching could still be observed on the fresh flush of *N. benthamiana* leaves at 65 dpi (Figure 2D). Although it is impossible to agroinfect cereals directly, *N. benthamiana* infiltrated leaves provide an excellent source for rub-inoculation to wheat. Leaf extracts from the ᐃCWMV:TaPDS (contains pCB-35S-R1 and pCB-35S-R3:TaPDS) and BSMV:TaPDS treatments caused systemic infection and symptoms when rub-inoculated to wheat. After 30 days, the PDS gene silencing phenotype was also observed in wheat (Figure 2C). Real-time RT-qPCR then showed that the mRNA levels of TaPDS and NbPDS were significantly less in the ᐃCWMV:TaPDS and ᐃCWMV:NbPDS treatments than in controls infected with the empty vector (ᐃCWMV:00) (Figure 2E). Thus, the modified CWMV vectors carrying NbPDS or TaPDS were effective in the silencing endogenous PDS in *N. benthamiana* and wheat.

### Optimizing the CWMV VIGS Vector in *N. benthamiana* and Wheat

**FIGURE 3:**
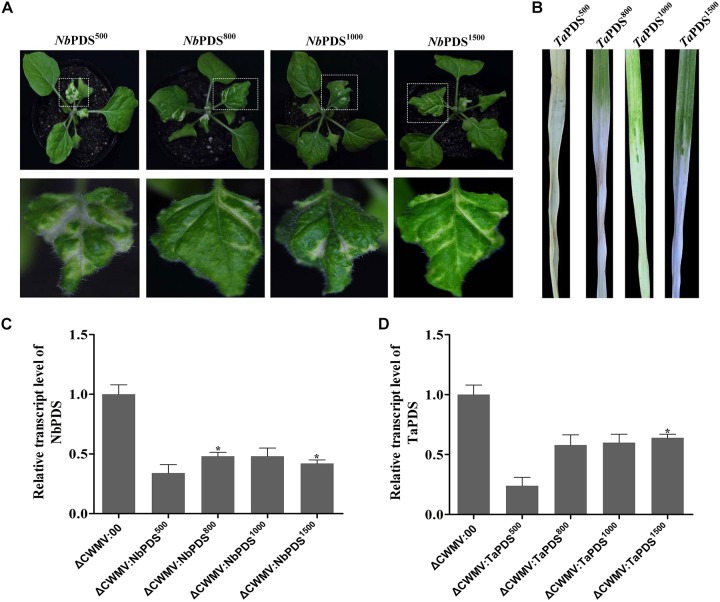
The effects of using PDS inserts of different lengths in *N. benthamiana* and *T. aestivum* plants inoculated with CWMV. **(A)** Representative *N. benthamiana* leaves 27 days post-inoculation with ᐃCWMV:*Nb*PDS^500^, ᐃCWMV:*Nb*PDS^800^, ᐃCWMV:*Nb*PDS^1000^, or ᐃCWMV:*Nb*PDS^1500^; bottom photograph is the enlargement of the boxed part of **(A)**. **(B)** Representative wheat leaves 42 days post-inoculation with ᐃCWMV:*Ta*PDS^500^, ᐃCWMV:*Ta*PDS^800^, ᐃCWMV:*Ta*PDS^1000^, or ᐃCWMV:*Ta*PDS^1500^. **(C)** Expression levels of PDS transcripts in *N. benthamiana* plants infected with ᐃCWMV:NbPDS^500^, ᐃCWMV:NbPDS^800^, ᐃCWMV:NbPDS^1000^, or ᐃCWMV:NbPDS^1500^. **(D)** Expression levels of PDS transcripts in wheat plants infected with ᐃCWMV:*Ta*PDS^500^, ᐃCWMV:*Ta*PDS^800^, ᐃCWMV:*Ta*PDS^1000^, or ᐃCWMV:*Ta*PDS^1500^. Bars represent the standard errors of the means. Three sample unequal variance directional *t*-test (^∗^*p* ≤ 0.05) was used to test the significance of the difference.

**FIGURE 4:**
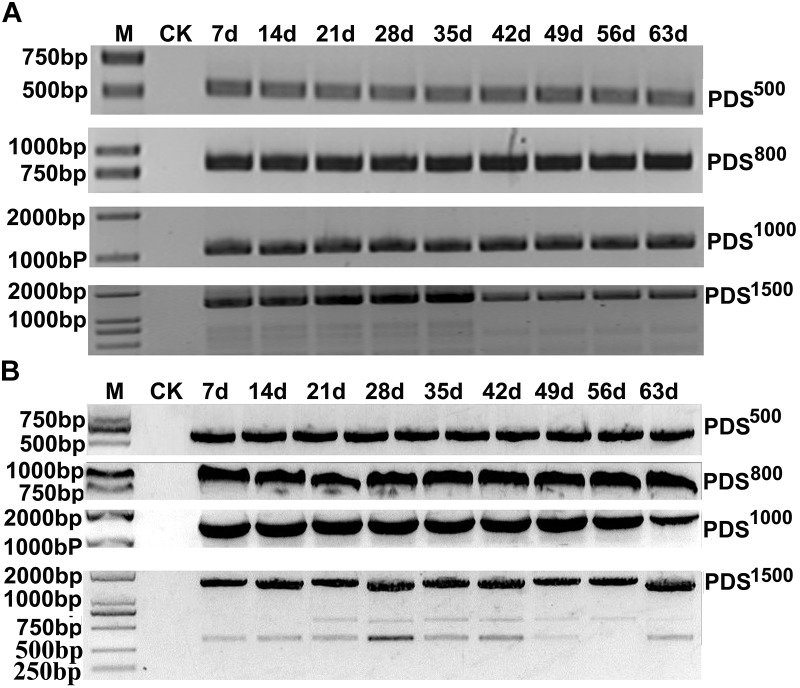
RT-PCR analysis of PDS insertion clones in MCS for insert stability. The samples were collected at 7 dpi (infiltrated leaf) and 14, 21, 28, 35, 42, 49, 56, and 63 dpi (systemic leaves) from *N. benthamiana* **(A)** and wheat **(B)**. RT-PCR products were separated on a 1.5% agarose gel with a 1 kB Plus DNA Ladder marker. Control amplifications from mock-inoculated sample RNAs (CK) are shown in each panel.

To determine the effects of fragment lengths on silencing efficiency, we assessed the phenotypes induced by 500, 800, 1000, and 1500 bp NbPDS and TaPDS fragments in *N. benthamiana* and wheat, respectively. Based on their photobleaching, 500 bp NbPDS inserts appeared to be the most efficient, and the 1500 bp NbPDS inserts were the least efficient after 27 dpi. It was then demonstrated by qPCR that the NbPDS RNA levels were most strongly reduced (to less than 15%) by the 500 bp NbPDS insertion (Figures 3A,C). Interestingly, the same result also was observed when PDS was VIGS in the wheat after 42 dpi (Figures 3B,D). For VIGS analyses, it is important to maximize foreign fragment stability in the infiltrated leaves and to minimize loss of inserts during virus movement. To evaluate the stability of inserts in infiltrated and systemically infected leaves, *N. benthamiana* and wheat leaves were infiltrated with PDS^500^, PDS^800^, PDS^1000^, or PDS^1500^ and samples were collected at 7 dpi from infiltrated leaves, and at 14, 21, 28, 35, 42, 49, 56, and 63 dpi from systemically infected *N. benthamiana* and wheat leaves, respectively. These were then tested by RT-PCR using primer pairs P14F/P14R covering the insertion site. The inserted sequence was relatively stable in the infiltrated leaves up to 63 dpi in most treatments, but distinct degradation products were observed in the PDS^1500^ treatment at all time points both in *N. benthamiana* and wheat (Figures 4A,B and Supplementary Figure S1).

### miRNA Activity Can Be Suppressed by Using the CWMV VIGS Vector to Express STTM Sequences in *N. benthamiana* and Wheat

**FIGURE 5:**
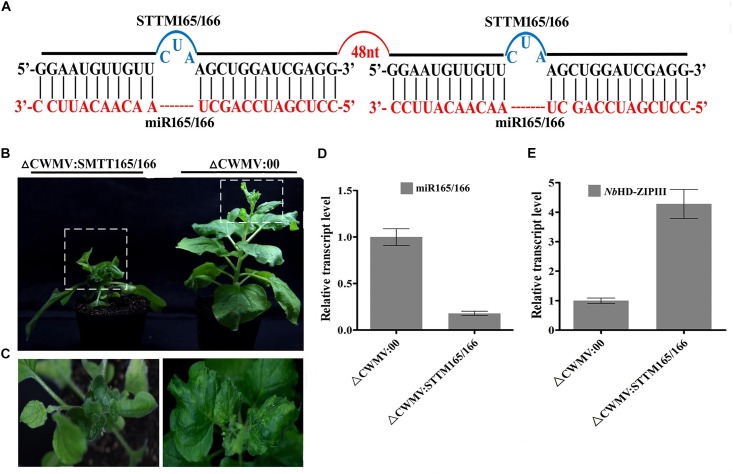
CWMV-based miR165/166 silencing using the STTM approach in *N. benthamiana*. **(A)** Diagrammatic representation of STTM165/166 structure. **(B)** *N. benthamiana* infected with ᐃCWMV:STTM165/166 (left) and with ᐃCWMV:00 (right) at 35 dpi. **(C)** Enlargement of the boxed parnt of **(B)**. **(D)** Stem-loop RT-qPCR detection of mature miR165/166 relative transcript levels in *N. benthamiana* infected with ᐃCWMV:00 or ᐃCWMV:STTM165/166. Error bars represent SE of three representative experiments from four replicates. **(E)** Real-time RT-qPCR analysis of mRNA levels of miR165/166 Target *Nb*HD-ZIPIII in ᐃCWMV:00 control and in plants expressing the STTM165/166 structure. Error bars show the SE calculated from three replicates.

**FIGURE 6:**
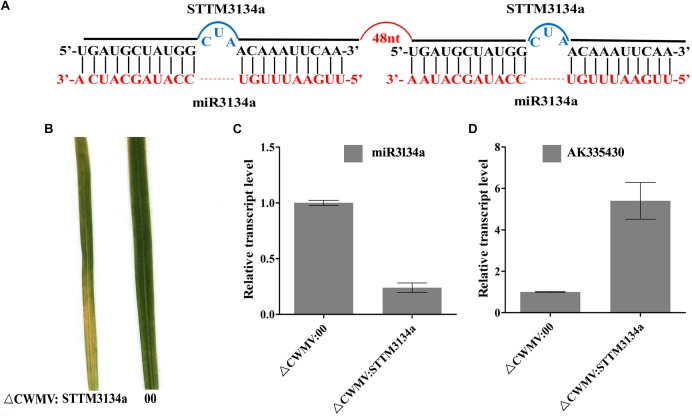
CWMV-based miR3134a silencing using the STTM approach in *T. aestivum*. **(A)** Diagrammatic representation of STTM3134a structure. **(B)** The fourth leaves of *T. aestivum* infected with ᐃCWMV:00 (left) and with ᐃCWMV:STTM3134a (right) at 20 dpi. **(C)** Stem-loop RT-qPCR detection of mature miR3134a relative transcript levels in *T. aestivum* infected with ᐃCWMV:00 or ᐃCWMV:STTM3134a. Error bars represent SE of three representative experiments from four replicates. **(D)** Real-time RT-qPCR analysis of mRNA levels of miR3134a target AK335430 in ᐃCWMV:00 control and in plants expressing target STTM3134a structure. Error bars show the SE calculated from three replicates.

To test whether the CWMV VIGS vector can also be used to inhibit miRNA activity, miR165/166 was cloned into pCB-35S-R3 to generate pCB-35S-R3: STTMmiR165/166 using the STTM strategy as previously described (Yan et al., 2012; Figure 5A). *N. benthamiana* plants infiltrated with ᐃCWMV:STTMmiR165/166 (contains pCB-35S-R1 and pCB-35S-R3: STTMmiR165/166) developed typical CWMV symptoms on their upper non-inoculated leaves (Figures 5B,C). Stem-loop RT-PCR together with real time PCR assays confirmed that the STTMmiR165/166 sequences were expressed in these plants (Figure 5D), that the transcript level of mature miR165/166 was reduced and that the expression of the Target gene Class III HOMEODOMAIN-LEUCINE ZIPPER transcriptional factors (HD-ZIPIII) mRNA was increased relative to ᐃCWMV:00 controls (Figure 5E). To determine whether the CWMV VIGS vector also can suppress the expression of miRNAs in wheat, miR3134 a novel miRNA of wheat was cloned into pCB-35S-R3 to obtain pCB-35S-R3:STTMmiR3134 using the STTM strategy (Figure 6A). After confirming that STTMmiR3134 sequences were expressed in the inoculated *N. benthamiana* leaves, wheat plants were rubbed with infiltrated *N. benthamiana* leaf sap. Wheat plants infiltrated with bbbCWMV:STTMmiR3134 developed etiolation symptoms on the fourth leaves at 20 dpi (Figure 6B). At 14 dpi, stem-loop RT-qPCR assays showed a decline in the relative transcript level of mature miR3134 in ᐃCWMV:STTMmiR3134-infected wheat (Figure 6C). RT-qPCR results also showed that the mRNA levels of the candidate target gene of miR3134, Genbank accession AK335430 (Jiao et al., 2015) were obviously increased compared to those in ᐃCWMV:00-infected wheat (Figure 6D).

### CWMV VIGS Vector Induce More Effective Gene Silencing Than Others at Low Temperature

**FIGURE 7:**
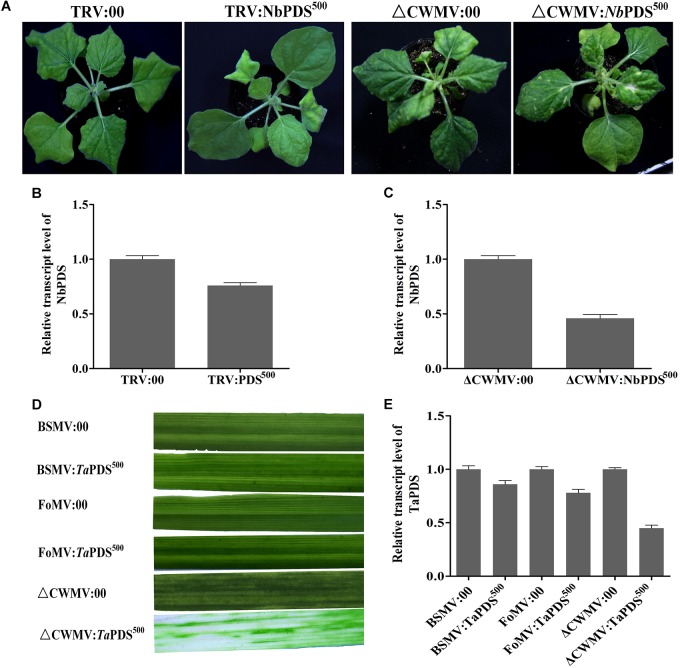
The effects of ᐃCWMV:PDS^500^ compare with TRV:PDS^500^, BSMV:PDS^500^, and FoMV:PDS^500^ in *N. benthamiana* or *T. aestivum* at 17°C, respectively. **(A)** Representative *N. benthamiana* leaves 27 dpi with TRV:*Nb*PDS^500^ or ᐃCWMV:*Nb*PDS^500^. **(B)** Real-time RT-qPCR analysis of mRNA levels of *Nb*PDS in TRV:00 control and in plants inoculated with TRV:*Nb*PDS^500^. Error bars show the SE calculated from three replicates. **(C)** Real-time RT-qPCR analysis of mRNA levels of *Nb*PDS in ᐃCWMV:00 control and in plants inoculated with ᐃCWMV:*Nb*PDS^500^. Error bars show the SE calculated from three replicates. **(D)** Representative of wheat leaves 42 dpi with BSMV:*Ta*PDS^500^, FoMV:*Ta*PDS^500^, or ᐃCWMV:*Ta*PDS^500^. **(E)** Real-time RT-qPCR analysis of mRNA levels of *Ta*PDS in wheat. The plants inoculated with BSMV:00, FoMV:00, or ᐃCWMV:00 as control, respectively. Error bars show the SE calculated from three replicates.

Virus-induced gene silencing vector CWMV- was compared with TRV- for inducing gene silencing in *N. benthamiana* and BSMV and FoMV in wheat. 500 bp NbPDS was selected and inserted into TRV and CWMV-based VIGS vector to construct TRV:NbPDS^500^ and CWMV:NbPDS^500^, respectively. After 27 dpi, the visible photobleaching could be observed on the fresh flush of *N. benthamiana* leaves induced by ᐃCWMV:NbPDS^500^ but not any white streaks appeared in the plants rubbed with TRV:NbPDS^500^ at 17°C (Figure 7A). RT-qPCR also showed that the amount of NbPDS mRNA was significantly less in the plants infected with ᐃCWMV:NbPDS^500^ than plants infected with TRV:*Nb*PDS^500^ (Figures 7B,C). Additionally, 500 bp TaPDS was selected and inserted into BSMV-, FoMV-, and CWMV-based VIGS vector to generate BSMV:TaPDS^500^, FoMV:TaPDS^500^ and CWMV:TaPDS^500^, respectively. Two-leaf-stage wheat plants were rubbed with saps of *N. benthamiana* leaves agroinfiltrated with BSMV:TaPDS^500^, FoMV:TaPDS^500^ or ᐃCWMV:TaPDS^500^, respectively. And these inoculated plants were maintained at 17°C as well. All wheat plants infected with ᐃCWMV:TaPDS^500^ appeared the bleached patches and streaks or albinism fully expanded in the fourth leaf at 42 dpi (Figure 7D). However, neither BSMV:TaPDS^500^ or FoMV:TaPDS^500^ inoculated plants showed any silencing phenotype (Figure 7D). To confirm the silencing of TaPDS genes, RT-qPCR was performed. The mRNA levels of TaPDS in the wheat rubbed with sap of ᐃCWMV:TaPDS^500^ were strikingly reduced compared to those of BSMV:TaPDS^500^ or FoMV:TaPDS^500^-infected plants (Figure 7E). Taken together, it clearly suggests that CWMV VIGS vector can induce more efficient gene silencing both in the *N. benthamiana* and wheat at 17°C.

## Discussion

Wheat is one of the most important staple crops in the world. As its draft genome has been sequenced and increasing numbers of genes have been annotated, a rapid system for transient/stable transformation is highly desirable to enable gene function analysis in this plant. Previous work showed that BSMV could be used to study wheat functional genomics (Holzberg et al., 2002) and subsequently the BSMV-mediated VIGS system has been extensively applied to study several functional genes and miRNAs in both dicots and monocots (Scofield et al., 2005; Loutre et al., 2009; Tufan et al., 2011; Jiao et al., 2015). Despite the effectiveness of this vector, it has intrinsic disadvantages. Not only is the VIGS phenotype superimposed on the chlorosis, leaf distortion and stunted growth symptoms of virus infection but BSMV has three genomic RNAs making experimental manipulation rather complex. By contrast, CWMV has only two genomic RNAs with a simple genomic structure and induces very mild symptoms in wheat, suggesting that it may have greater potential as a VIGS vector in wheat and *N. benthamiana*.

As previous studies on furoviruses have shown the coat protein read through region is prone to spontaneous deletions during prolonged infection or mechanical virus transmission. The virus remains fully infectious as this region is not required for viral encapsidation, replication, and systemic infection (Yang et al., 2016) but probably plays a role in viral transmission through soil-borne zoospores (Yamamiya et al., 2005). This read through region is therefore an obvious choice for sequence insertions. The pCB-35S-R3 deletion mutant was able to replicate and systemically infect *N. benthamiana* (Figure 1B) and wheat (Figure 1D). When partial PDS gene sequences derived from *N. benthamiana* and wheat were inserted into the vector to generate pCB-35S-R3:NbPDS and pCB-35S-R3:TaPDS, they remained infectious. pCB-35S-R3:*Nb*PDS induced visible photobleaching in *N. benthamiana* but pCB-35S-R3:TaPDS did not (Figure 2B), although typical photobleaching occurred on the leaves of wheat rub-inoculated with pCB-35S-R3:TaPDS-infiltrated *N. benthamiana* leaf extracts (Figure 2C). This is probably explained by previous research that suggests that RNA silencing only occurs where expressed sequences are more than 80% identical to the target gene (Burton et al., 2000; Xu et al., 2001). The size of the insert sequence may affect efficiency of target gene silencing (Avesani et al., 2007) and in our studies the length of the inserted PDS fragment between 500 and 1500 bp affected the degree of photobleaching (Figures 3A,B) and there was a 70 to 84% reduction in PDS mRNA compared with plants inoculated with CWMV:00 not only in the *N. benthamiana* but also in the wheat (Figures 3C,D). Large inserts may also be lost more frequently giving short-lasting silencing compared with smaller inserts (Avesani et al., 2007) and our results also showed that the longest insert was less stable than the others (Figure 4). Some researchers suggest that the insert stability may be affected by the sequence of the insert (Zhong et al., 2005). Our investigation only investigated the stability of PDS silencing and does not exclude the possibility that other Target genes may show different kinetics of silencing. Interestingly, and in contrast to CWMV:NbPDS, no significant photobleaching was observed in the leaves of *N. benthamiana* inoculated with TRV:NbPDS at 17°C (Figure 7A). The same situation also was observed in the wheat inoculated with FoMV:TaPDS or BSMV:TaPDS at 17°C (Figure 7D). However, the results of RT-PCR showed TRV, BSMV and FoMV have caused systemic infection in inoculated plants (Supplementary Figure S2). There are several possible reasons for this. First, 17°C is the optimum temperature for CWMV infection (Yang et al., 2016) but not for TRV, FoMV, and BSMV (Ratcliff et al., 2001; Bruun-Rasmussen et al., 2007; Liu et al., 2016). Second, the PDS silencing efficiency was positively correlated with photobleaching phenotype in plant (Bruun-Rasmussen et al., 2007). Indeed, although RT-PCR detection showed that all of the TRV, BSMV, and FoMV could cause systemic infection in the inoculated plants (Supplementary Figure S1), the real time RT-PCR showed the mRNA level of PDS was modestly reduced in the plants infected by TRV-NbPDS, BSMV:TaPDS, or FoMV:TaPDS (Figures 7B,E). In addition, temperature as well as insert size may influence the stability of inserts carried by a recombinant virus (Qu et al., 2005).

Recently, there has been increasing interest in exploring the function of plant miRNAs and several experimental approaches have been used in different plant species (Sha et al., 2014; Chen et al., 2015; Liao et al., 2015). The STTM strategy simultaneously blocks several different miRNAs and is particularly useful (Yan et al., 2012). For our study, we selected two contrasting miRNA targets miRNA165/166 is highly expressed and conserved in monocot and dicot plants while miR3134a is specific to wheat and barley and is expressed at lower levels in wheat leaves during different development stages. Our results indicated that the CWMV-based miRNA silencing system over expressing miRNA target mimics was efficient in blocking endogenous miRNAs in wheat.

We have therefore demonstrated the utility of CWMV for silencing endogenous genes or miRNAs of *N. benthamiana* and wheat in vegetative tissues. Although, the capacity of CWMV for carrying and expressing foreign genes needs further exploration, a wide application of CWMV as a VIGS vector for endogenous gene silencing seems quite possible. Our data also clearly displayed the CWMV-based VIGS system is more efficient than TRV-, BSMV-, and FoMV-based VIGS system at 17°C. Thus, the modified CWMV-based silencing system has great potential for functional characterization of target genes in monocots and dicots at low temperature.

## Materials and Methods

### Plant Material and Growth Conditions

*Nicotiana benthamiana* plants were grown in a cabinet at 17°C, with 16 h light/8 h dark and 70% r.h. Wheat (Yangmai 158) plants used for VIGS experiments were grown in a greenhouse until the four leaf-stage, then inoculated with CWMV and transferred to a climate chamber at 17°C until evaluation.

### Molecular Cloning and Plasmid Construction

pCB-35S-R1 and pCB-35S-R2 are full-length cDNA clones of RNA1 and RNA2 of CWMV developed and stored in our lab. *Escherichia coli* DH5α was used for the construction of all plasmids and all plasmids constructed in this study were verified by sequencing. The primers used in this paper are detailed in Supplementary Table S1.

pCB-35S-R3 was constructed as follows. The mutant construct pCB-35S-R2:M5 which abolished the translation of the N-terminal extension of CP and the RT domain of the CP was developed and stored in our lab and was selected as template. The primer pair P1F/P1R was used to amplify the segment S1 from pCB-35S-R2:M5. This 550 bp PCR product was digested with *Spe I/Sal I* and ligated into the similarly digested plasmid pCB-35S-R2:M5 to obtain pCB-35S-R3 containing a MCS.

*Chinese wheat mosaic virus* gene silencing constructs were prepared as follows. The *N. benthamiana* 300, 500, 800, 1000, and 1500 bp PDS fragments (NbPDS; Genbank accession: EU165355) were obtained by RT-PCR using the P2F/P2R, P3F/P3R, P4F/P4R, P5F/P5R, and P6F/P6R primer pairs (Supplementary Table S1), respectively. The resulting fragments were integrated into pCB-35S-R3 in the sense orientation to generate pCB-35S-R3:NbPDS^300^, pCB-35S-R3:NbPDS^500^, pCB-35S-R3:NbPDS^800^, pCB-35S-R3:NbPDS^1000^, and pCB-35S-R3:NbPDS^1500^, respectively. In a similar fashion, 300 bp *Triticum aestivum* PDS fragments (TaPDS; Genbank accession: FJ517553) were amplified by RT-PCR with P7F/P7R primer pairs (Supplementary Table S1) and inserted into pCB-35S-R3.

STTM165/166 (for silencing of miR165/166 using the STTM strategy) was constructed as follows. A primer with restriction enzyme cutting site, corresponding to the target mimic of miR165/166 and 48 nt STTM spacer (5′-GTTGTTGTTGTTATGGTCTAATTTAAATATGGTCTAAAGAAGAAGAAT-3′) were used to amplify STTM165/166by PCR using primers P8F/P8R (Figure 5A). The resulting fragments were integrated into pCB-35S-R3 to generatepCB-35S-R3:STTM165/166. STTM3134a (for silencing of miR3134a) was constructed by the same method using primers P9F/P9R (Figure 6A).

### Northern Blot Assays

Northern blot assays were performed as previously described (Yang et al., 2016). Total RNAs were extracted from leaf tissues using TRIzol reagent (Invitrogen) and treated with DNase (TaKaRa). Three micrograms of total RNAs were separated on a denaturing 2% formaldehyde agarose gel and transferred to Hybond-N^+^ membranes (Amersham Bioscience) using 20× SSC buffer. The RNAs were cross-linked to membrane matrix by UV for 50 s. Northern blotting for assays of CWMV genomic RNAs was carried out using the DIG High Prime DNA Labeling and Detection Starter Kit II (Roche). The DNA oligo nucleotides complementary to the 3′ terminus of the CWMV genome were labeled with digoxigenin (DIG) at their 3′ ends using the DIG Oligo nucleotide Tailing Kit (Roche) and then purified using a G25 Sephadex column (GE). Membranes were pre-hybridized for 2 h and hybridized overnight at 42°C using the DIG Luminescent Detection Starter kit for nucleic acids (Roche). The hybridization signals were visualized by Amersham Imager 600 (GE). All these procedures were performed according to the manufacturers’ instructions.

### Agroinfiltration of *N. benthamiana* and Viral Inoculation of Cereals

The pCB-35S-R3 derivatives were transformed into *A. tumefaciens* strain GV3101. For agroinfiltration, single colonies were cultured overnight at 28°C with constant shaking in 10 ml of LB containing kanamycin (100 μg/ml) and rifampicin (25 μg/ml). Then, 0.1 ml aliquots were used to inoculate 10 ml LB containing the same antibiotics and grown at 28°C for 10 h. Bacterial cells were pelleted at 5000 rpm for 5 min, resuspended in infiltration buffer containing 10 mM 2-(*N-morpholino*) ethanesulfonic acid (MES), pH 5.2, 10 mM MgCl_2_, and 0.1 mM acetosyringone to 0.6–0.7 OD_600_ and incubated at room temperature for 2–3 h. For agroinfiltration, equal amounts of bacteria harboring pCB-35S-R1 and pCB-35S-R3 (or its derivatives) were mixed, and infiltrated into four to six *N. benthamiana* leaves immediately above the cotyledons with a 1 ml needleless syringe. After maintenance in a growth chamber for 7–10 days post infiltration (dpi), the infiltrated leaves were harvested, ground in 20 mM Na-phosphate buffer (pH 7.2) containing 1% celite, and the sap was mechanically inoculated onto wheat seedlings at the two-leaf stage.

### RNA Extraction and PCR Analysis

Total RNAs were extracted from three independent biological replicates of *N. benthamiana* plants, uninoculated wheat plants, CWMV-infected *N. benthamiana* plants, and CWMV-infected wheat plants with TRIzol reagents as described by the manufacturer (Invitrogen). Real-time quantification of microRNAs was performed by stem-loop RT-qPCR as previously reported (Salone and Rederstorff, 2015). Briefly, 2 μg purified total RNA were reverse transcribed using miRNA-specific stem-loop primers (50 nM) in a 20-μl reaction volume with the Takara RNA PCR Kit to generate cDNA following the manufacturer’s instructions. The expression patterns of the miRNAs were then analyzed by real-time quantitative PCR (qPCR) with a standard SYBR Green I PCR kit (Invitrogen) protocol. Each qPCR reaction contained 10 μl of 2 × SYBR PCR master mix, 0.8 μl of forward primer (10 μM), 0.8 μl of reverse primer (10 μM), 0.5 μl of cDNA, and sterilized ddH_2_O added up to 20 μl. The reactions were incubated in a 96-well plate at 95°C for 10 min, followed by 40 cycles of 95°C for 15 s and 60°C for 1 min. Then, the samples were heated from 60 to 95°C to acquire the denaturing curve of the amplified products. All reactions were performed in triplicate on a 7900 Real Time PCR System (Applied Biosystems). Relative expression was calculated using the comparative cycle threshold method (Livak and Schmittgen, 2001; Schmittgen and Livak, 2008) and values for each miRNA were normalized to the expression levels of U6. All the primers used are listed in Supplementary Table S1.

The expression of the Target gene was also analyzed by qPCR. The total RNA was first treated with DNase I (Takara) and then reverse transcribed to generate cDNA using an oligo (dT) primer and a Primescrip RT reagent kit (Takara) according to the manufacturer’s instructions. The primer pair P14F/P14R (see Supplementary Table S1) was then added to amplify the PCR products. The qPCR was performed in triplicate on a 7900 Real Time PCR System (Applied Biosystems) with a standard SYBR Green I PCR kit (Invitrogen) protocol. In each reaction, actin gene for which stabilized expression after virus infection was used as the internal reference (Supplementary Figure S3). The reactions were incubated in a 96-well plate at 95°C for 3 min, followed by 40 cycles of 95°C for 15 s, 60°C for 20 s, and 72°C for 30 s. Each experiment was replicated three times. The relative expression levels were calculated using the 2^-ΔΔCT^ method (Livak and Schmittgen, 2001). The values of threshold cycle (CT) were calculated by Rotor-Gene 6 software (Corbett Robotics, Australia) and the 2^-ΔΔCT^ method was used to assess the relative changes in gene expression (Nobuta et al., 2010).

## Author Contributions

H-MZ and J-PC were responsible for study conception, design, and coordination. JiY, LH, T-YZ, J-BL, Q-SL, and JL carried out the experiments for vector construction, northern blotting, real-time RT-PCR, and VIGS assays. LH, T-YZ, and JaY performed the virus inoculation, agro-infiltration, sample collection, and RNA isolation for gene expression and blotting assays. H-MZ, JaY, and J-PC were responsible for data collection and analysis and drafted the manuscript. All authors read and approved the final manuscript.

## Conflict of Interest Statement

The authors declare that the research was conducted in the absence of any commercial or financial relationships that could be construed as a potential conflict of interest.

## Supplementary Material

FIGURE S1 RT-PCR analysis of different size of PDS insertion clones in MCS for insert stability in *N. benthamiana* **(A)** and *T. aestivum* **(B)**.

FIGURE S2 RT-PCR detection of plants inoculated with TRV, CWMV, BSMV, and FoMV, respectively.

FIGURE S3 Semi-quantitative analysis the stability of actin reference gene after virus infection.

TABLE S1 Primers used in vector construction and PCR analysis.
