# Supplementary figures and images for: Chinese Wheat Mosaic Virus-Induced Gene Silencing in Monocots and Dicots at Low Temperature
Source: Front Plant Sci. 2018 Nov 14;9:1627. doi: 10.3389/fpls.2018.01627 (PMC6247046; doi:10.3389/fpls.2018.01627)

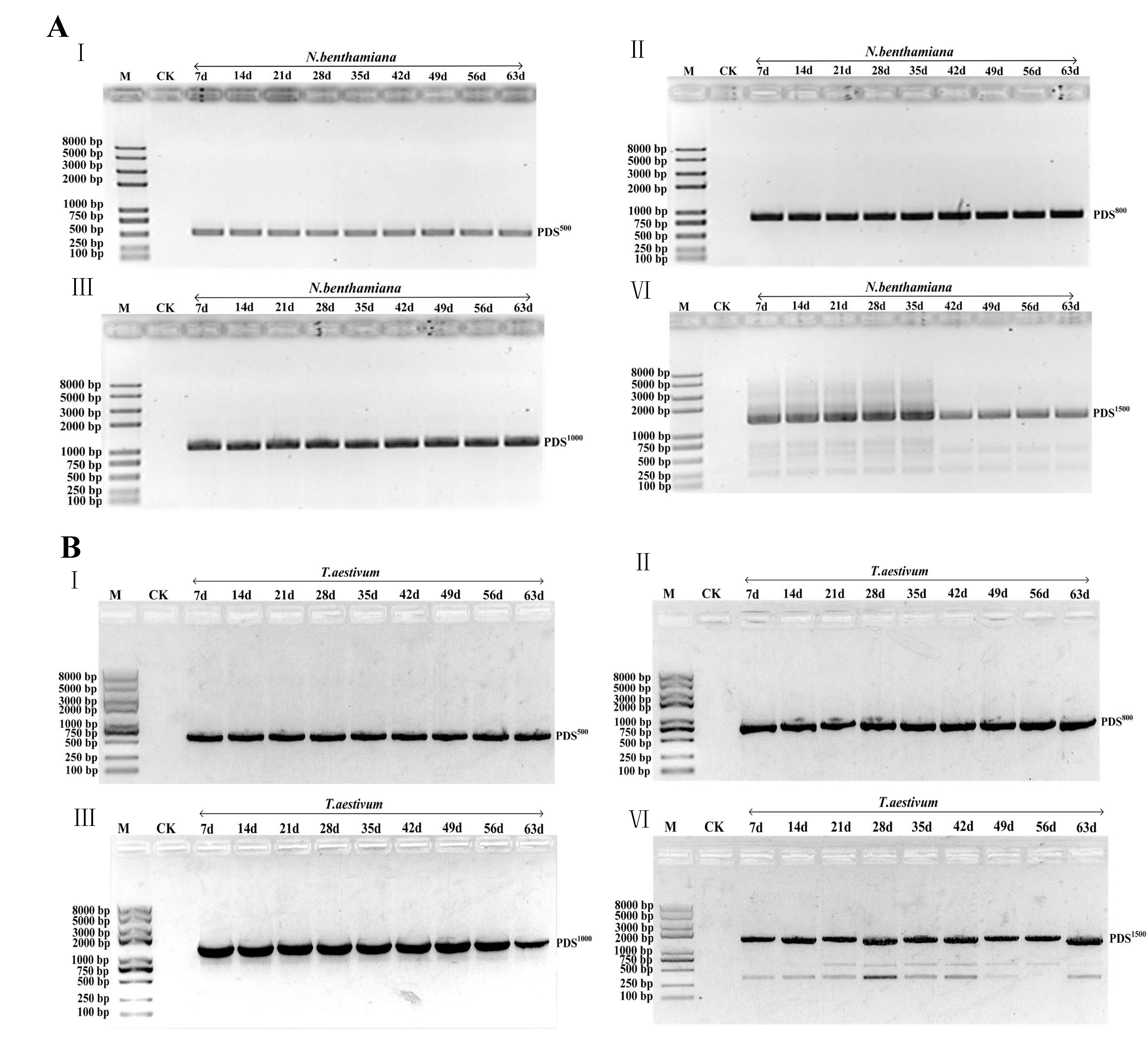

Supplement: FIGURE S1 — RT-PCR analysis of different size of PDS insertion clones in MCS for insert stability in N. benthamiana (A) and T. aestivum (B). [file Image_1.JPEG]

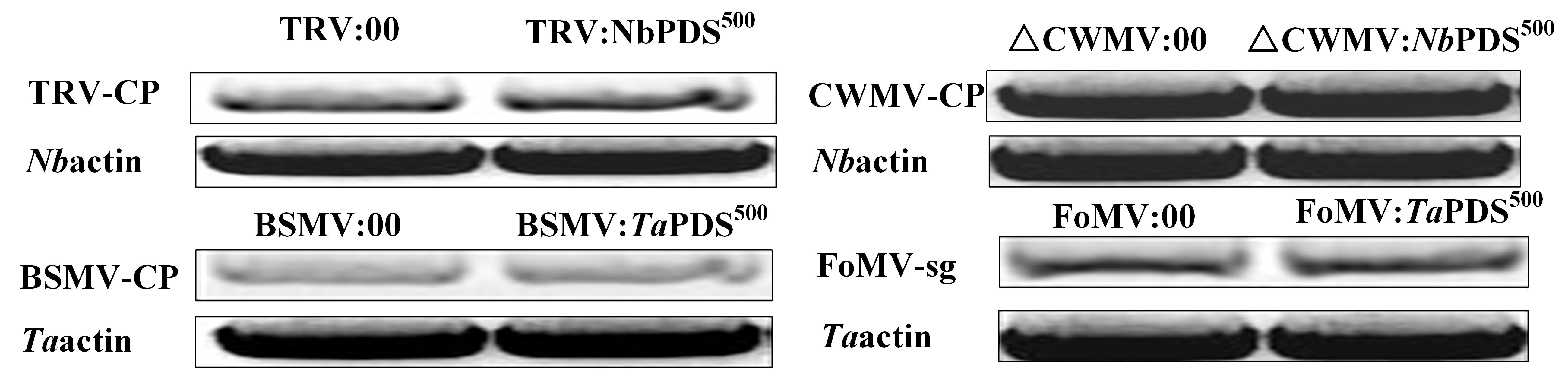

Supplement: FIGURE S2 — RT-PCR detection of plants inoculated with TRV, CWMV, BSMV, and FoMV, respectively. [file Image_2.JPEG]

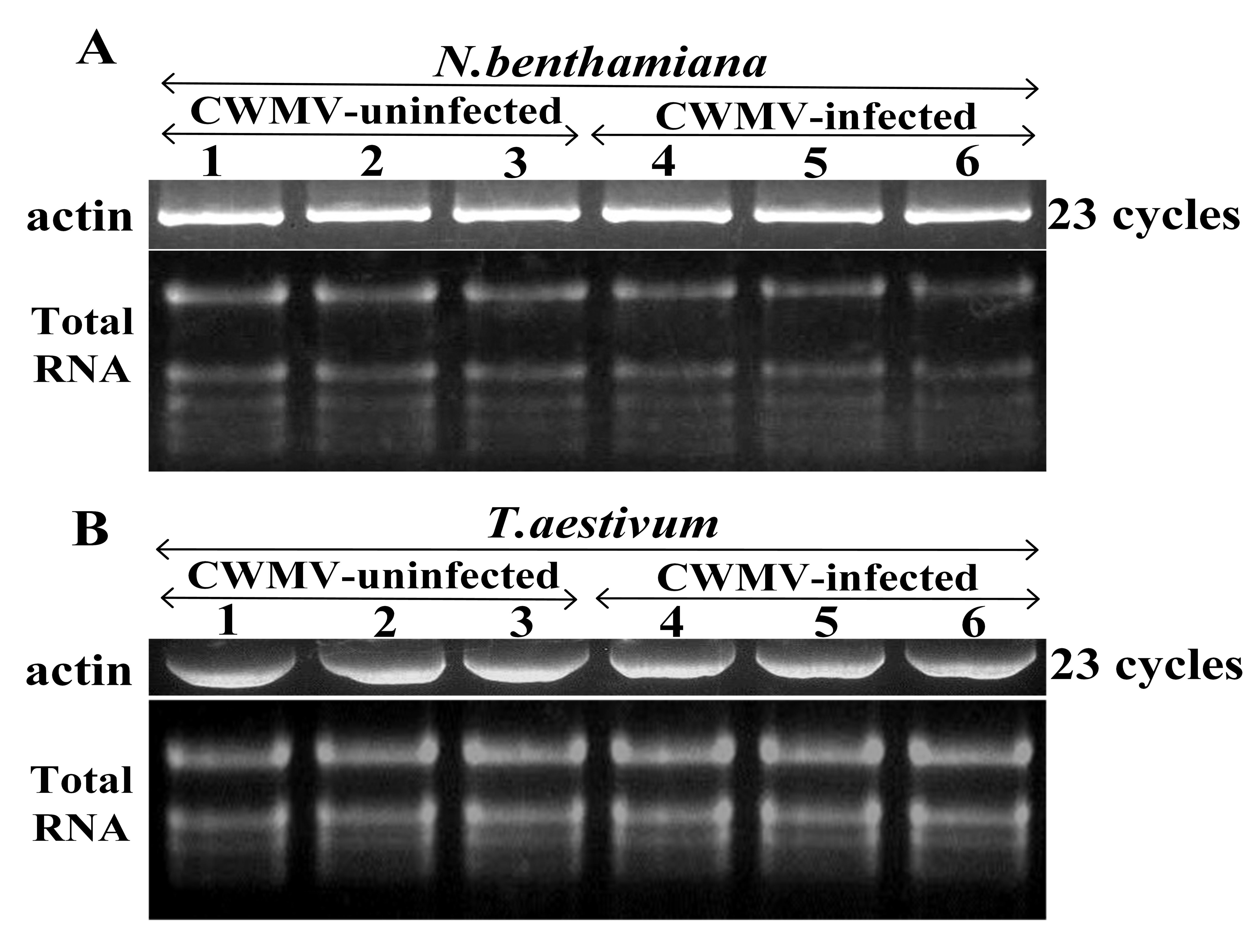

Supplement: FIGURE S3 — Semi-quantitative analysis the stability of actin reference gene after virus infection. [file Image_3.JPEG]
