# Supplementary material for: Chinese Wheat Mosaic Virus-Induced Gene Silencing in Monocots and Dicots at Low Temperature
Source: Front Plant Sci. 2018 Nov 14;9:1627. doi: 10.3389/fpls.2018.01627 (PMC6247046; doi:10.3389/fpls.2018.01627)
Supplement: TABLE S1 — Primers used in vector construction and PCR analysis. [file Table_1.docx]

**Table S1. Primers used in vector construction and PCR analysis.**

| **Number** | **Primers** | **Primer sequences (5’-3’)** |
| --- | --- | --- |
| P1F | R3MCS-F^a,b^ | 5’- **G**ACTAGTTGGTACCACGCGTGGTGGGAAAAGTGGTGTGAGTAG -3’ |
| P1R | R3MCS-R^b^ | 5’- ATTTTTTCGTCGACATTGAAGG-3’ |
| P2F | R3NbPDS^300^-F^b^ | 5’- **G**ACTAGTATTTACTGCTATCTTGTTCAA-3’ |
| P2R | R3NbPDS^300^-R^b^ | 5’-**GC**ACGCGTCAAATAGTTAACTGTATTGTC-3’ |
| P3F | R3NbPDS^500^-F^b^ | 5’- **G**ACTAGTATTTACTGCTATCTTGTTCAA-3’ |
| P3R | R3NbPDS^500^-R^b^ | 5’-**GC**ACGCGTCCAATCTCCA TCATCATCTTT-3’ |
| P4F | R3NbPDS^800^-F^b^ | 5’- **G**ACTAGTATTTACTGCTATCTTGTTCAA-3’ |
| P4R | R3NbPDS^800^-R^b^ | 5’- **GC**ACGCGTACCGTCTTGA GCTTCAACATA-3’ |
| P5F | R3NbPDS^1000^-F^b^ | 5’- **G**ACTAGTATTTACTGCTATCTTGTTCAA-3’ |
| P5R | R3NbPDS^1000^-R^b^ | 5’- **GC**ACGCGTCTCTCAGGAG GGTTACCATCT-3’ |
| P6F | R3NbPDS^1500^-F^b^ | 5’- **G**ACTAGTATTTACTGCTATCTTGTTCAA-3’ |
| P6R | R3NbPDS^1500^-R^b^ | 5’- **GC**ACGCGTTGCCGAAATTTCATCAGGGAA-3’ |
| P7F | R3TaPDS^300^-F^b^ | 5’-**G**ACTAGTATGGATACCAGCTGCCTATCA-3’ |
| P7R | R3TaPDS^300^-R^b^ | 5’-**GC**ACGCGTAGTGAACGCCCCAGTAAACCAT-3’ |
| P8F | STTM165/166-F^b,C^ | 5’-**G**ACTAGT***CCTCGATCCA GCTAACAACATTCC***GGTCTAATTTAAATATGGTCTAAAGAAGAAGAAT-3’ |
| P8R | STTM165/166-R^b,C^ | 5’-**GC**ACGCGT***GGAATGTTGTTAGCTGGATCGAGG***ATTCTTCTTCTTTAGACCATATTTAAATTAGACC-3’ |
| P9F | STTM3134a-F^b,C^ | 5’-**G**ACTAGT***TGATGCTATGGCTAACAAATTCAA*** GGTCTAATTTAAATATGGTCTAAAGAAGAAGAAT-3’ |
| P9R | STTM3134a-R^b,C^ | 5’-**GC**ACGCGT***TTGAATTTGTTAGCCATAGCATCA***ATTCTTCTTCTTTAGACCATATTTAAATTAGACC-3’ |
| P10F | U6-F | 5’-GGGGACATCCGATAAAATTGG-3’ |
| P10R | U6-R | 5’-GGACCATTTCTCGATTTGTGC-3’ |
| P11F | Actin-F | 5’-TGGCACCCGAGGAGCACC-3’ |
| P11R | Actin-R | 5’-GTAACCTCTCTCGGTGAG-3’ |
| P12F | micRNA165/166-F^d^ | 5’-CGGCGGGAATGTTGTTAGCTGGA-3’ |
| P12R | micRNA165/166-SL^e^ | 5’-GTCGTATCCAGTGCAGGGTCCGAGGTATTCGCACTGGATACGAGGAATG-3’ |
| P13F | micRNA3134a-F^d^ | 5’-GCCGCTTGAATTTGTCCATA-3’ |
| P13R | micRNA3134a-SL^e^ | 5’-GTCGTATCCAGTGCAGGGTCCGAGGTATTCGCACTGGATACGACTGATGC-3’ |
| P14F | CWMVR2^1000-1021^-F ^f^ | 5’-ACTAGAAGACAGATTACTGAG-3’ |
| P14R | CWMVR2^2600-2579^-R ^f^ | 5’-AGATCGCCAAAGGCTTACTAG-3’ |

^a^, the protective base is in bold font; ^b^, the restriction endonuclease is underlined; ^c^, the STTM mimic sequence is bold and iTalicized; ^d^, miRNA sequence is underlined; ^e^, Reverse-complement sequence of miRNA is underlined; ^f^, a pair of primers beyond the insertion site.
